# P2X7 receptor-pannexin 1 interaction mediates extracellular alpha-synuclein-induced ATP release in neuroblastoma SH-SY5Y cells

**DOI:** 10.1007/s11302-017-9567-2

**Published:** 2017-05-17

**Authors:** Anna Wilkaniec, Magdalena Gąssowska, Grzegorz A. Czapski, Magdalena Cieślik, Grzegorz Sulkowski, Agata Adamczyk

**Affiliations:** 1 Department of Cellular Signalling, Mossakowski Medical Research Centre, Polish Academy of Sciences, Pawińskiego 5 St., 02-106 Warsaw, Poland; 2 Department of Neurochemistry, Laboratory of Pathoneurochemistry, Mossakowski Medical Research Centre, Polish Academy of Sciences, Pawińskiego 5 St., 02-106 Warsaw, Poland

**Keywords:** Alpha-synuclein, Purinergic P2 receptors, P2X7 receptor, Extracellular ATP, Ecto-ATPase, Pannexin 1

## Abstract

Abnormalities of alpha-synuclein (ASN), the main component of protein deposits (Lewy bodies), were observed in Parkinson’s disease (PD), dementia with Lewy bodies, Alzheimer’s disease, and other neurodegenerative disorders. These alterations include increase in the levels of soluble ASN oligomers in the extracellular space. Numerous works have identified several mechanisms of their toxicity, including stimulation of the microglial P2X7 receptor leading to oxidative stress. While the significant role of purinergic signaling—particularly, P2 family receptors—in neurodegenerative disorders is well known, the interaction of extracellular soluble ASN with neuronal purinergic receptors is yet to be studied. Therefore, in this study, we have investigated the effect of ASN on P2 purinergic receptors and ATP-dependent signaling. We used neuroblastoma SH-SY5Y cell line and rat synaptoneurosomes treated with exogenous soluble ASN. The experiments were performed using spectrofluorometric, radiochemical, and immunochemical methods. We found the following: (i) ASN-induced intracellular free calcium mobilization in neuronal cells and nerve endings depends on the activation of purinergic P2X7 receptors; (ii) activation of P2X7 receptors leads to pannexin 1 recruitment to form an active complex responsible for ATP release; and (iii) ASN greatly decreases the activity of extracellular ecto-ATPase responsible for ATP degradation. Thus, it is concluded that purinergic receptors might be putative pharmacological targets in the molecular mechanism of extracellular ASN toxicity. Interference with P2X7 signaling seems to be a promising strategy for the prevention or therapy of PD and other neurodegenerative disorders.

## Introduction

Both oligomerization and accumulation of alpha-synuclein (ASN) are the key molecular processes involved in the pathophysiology of neurodegenerative diseases such as Parkinson’s disease (PD), Alzheimer’s disease (AD), and other synucleinopathies [1]. Alterations of ASN expression and impairment of its degradation can lead to the formation of intracellular deposits of this protein, called Lewy bodies (LB) [2]. Previous data have indicated that the key process responsible for the propagation and expansion of neurodegeneration in the brain is the release of ASN to extracellular space [1, 3]. According to these, ASN released to extracellular compartment (by exocytosis or as an effect of neurodegeneration) easily changes the structure to β-sheet and acts outside the cell or penetrates the neuronal or glial cells in a manner dependent on the concentration and aggregation stage [4]. When ASN reaches the increased concentration inside the neuron, oligomerization that leads to cell death starts. The molecular mechanisms of extracellular ASN-mediated toxicity include Ca^2+^ deregulation, enhancement of the reactive oxygen and nitrogen species production, mitochondria dysfunction, deregulation of dopaminergic and glutamatergic neurotransmission, and activation of brain neuroinflammation [5–12].

In parallel to the ASN-related hypothesis for neurodegenerative disorders, extracellular ATP release and purinergic neurotransmission have been recently shown to participate in the pathogenesis and progression of nervous system diseases [13, 14]. A significant amount of ATP can be released from many cell types in response to mechanical stress, inflammation, oxygen deprivation, or apoptotic stimuli through the opening of pannexin 1 channels [15–18]. In addition, ATP could be stored in special secretory vesicles and released by exocytosis from nerve terminals [19]. The secreted ATP is later sequentially degraded to ADP, AMP, and adenosine by plasma membrane nucleotidases [20, 21]. Activity of ATP and other extracellular nucleotides (ADP, UTP, and UDP) is mediated by the membrane P2 receptors, which based on pharmacology [22] and molecular cloning [23] can be subdivided into two types—ionotropic (P2X) and metabotropic (P2Y) receptors. Most studies of the extracellular actions of ATP connected with the short-term neurotransmission and neuromodulation events are related to P2X receptor-mediated Ca^2+^ permeability and membrane depolarization. However, by acting on P2 receptors, ATP can also have potent long-term (trophic) role by regulating two important second messengers: cytoplasmic Ca^2+^ and cyclic adenosine monophosphate (cAMP) [24, 25], thus providing a direct link between functional activity in neural circuits and cellular growth and differentiation. Depending on the cell type, expression of selective receptor subtypes, ecto-enzymes activity, and functional state of the cells, purinergic signaling may activate various signaling pathways from mitogenesis to apoptosis [24]. In pathological conditions, like cerebral trauma, ischemia, or neurodegeneration, not only the extracellular release of nucleotides may be significantly elevated, but also the expression of P2 receptors and the activity of extracellular ecto-nucleotidases may undergo certain changes, resulting in CNS dysfunctions [26]. Currently, it is being emphasized that purinergic signaling-dependent induction of neuroinflammation plays an important role in pathophysiology and progression of neurodegeneration [27–29]. Extracellular nucleotides regulate inflammatory signaling by inducing the microglia and astrocytes activation [30, 31], cytokines release (IL-1β, IL-6, TNF-α), and phagocytosis [32]. In the course of neurodegeneration, the change in P2 receptor expression and activity in neuronal cells was also observed. The P2X7 receptor-mediated Ca^2+^ entry and mitochondrial dysfunction were previously observed to play important role in the ATP-induced neuronal death [33]. Release of ATP from disrupted cells might cause cell death in neighboring cells, which express P2X7 receptors, leading to a necrotic volume increase [34]. Based on these data, deregulation of purinergic signaling may be an important factor related to ASN-induced pathology within CNS. Jiang et al. [12] showed that stimulation of the microglial P2X7 receptor by extracellular ASN resulted in increased oxidative stress. Although this interaction is relevant, it is yet to be studied within the neuronal cells. Therefore, the aim of this project was to investigate whether ASN can also induce changes in the ATP-mediated signaling in neuronal cells.

## Materials and methods

### Materials

ASN was obtained from rPeptide (Bogart, GA, USA). Antagonists of purinergic receptors, such as 3-[1-[[(3′-nitro[1,1′-biphenyl]-4-yl)oxy]methyl]-3-(4-pyridinyl)propyl]-2,4-thiazolidinedione (AZ 11645373), 5-(3-bromophenyl)-1,3-dihydro-2H-benzofuro[3,2-*e*]-1,4-diazepin-2-one (5-BDBD), and (1R*,2S*)-4-[2-chloro-6-(methylamino)-9H-purin-9-yl]-2-(phosphonooxy)bicyclo[3.1.0]hexane-1-methanol dihydrogen phosphate ester diammonium salt (MRS 2279) were obtained from Tocris Bioscience (Bristol, UK). Neuroblastoma SH-SY5Y cell line and cell culture reagents, such as minimum essential medium eagle (MEM), Ham’s F12 medium, Hank’s balanced salt solution (HBSS), non-essential amino acid solution, fetal bovine serum (FBS), penicillin, streptomycin, and l-glutamine, as well as antibodies anti-P2X7R, anti-P2Y1, anti-glyceraldehyde 3-phosphate dehydrogenase (GAPDH), anti-rabbit IgG, and other reagents such as ARL 67156, ATP disodium salt, carbenoxolone (CBX), pyridoxal-5′-phosphate-6-azo-phenyl-2,4-disulfonate (PPADS), TRI-reagent, DNase I, dimethyl sulfoxide (DMSO), and bovine serum albumin (BSA) were purchased from Sigma-Aldrich (St. Louis, MO, USA). Reagents for reverse transcription (high capacity RNA-to-complementary DNA (cDNA) Master Mix), PCR (Gene Expression Master Mix), TaqMan Gene expression assays, Fluo-4 AM, and Molecular Probes® ATP determination kit were obtained from Thermo Fisher Scientific (Waltham, MA, USA). Clarity™ Western ECL Substrate was from Bio-Rad Laboratories (Hercules, CA, USA). Cell lysis buffer was obtained from Cell Signaling Technology (Beverly, MA, USA). All other reagents were purchased from POCh (Gliwice, Poland).

### Preparation of soluble ASN

Human ASN was dissolved in phosphate-buffered saline (PBS) (pH 7.4) at a concentration of 100 μM and immediately used for experiments as soluble ASN in the form of mixture of monomers and oligomers [9].

### Cell culture

The studies were carried out using human neuroblastoma SH-SY5Y cell line, which is known to be able to both proliferate and differentiate in culture. SH-SY5Y cells were cultured in F12/MEM medium supplemented with 15% heat-inactivated FBS, 1% non-essential amino acids, 50 units/ml penicillin, and 50 μg/ml streptomycin as well as l-glutamine at 37 °C in a humidified incubator containing 5% CO_2_.

### Cellular treatment

SH-SY5Y cells were plated in 60- and 35-mm culture dishes or 96-well plates, and the growth medium was changed into a low-serum medium (MEM/F12 supplemented with 2% FBS, 1% penicillin/streptomycin, and 1% l-glutamine). HBSS or other media appropriate for the particular procedure were also used. Then, the cells were treated with exogenous ASN (10 μM), specific agonist, and antagonists of purinergic receptors: ATP (5 mM, pH 7.3–7.5), PPADS (100 μM, dissolved in H_2_O), MRS 2279 (10 μM, dissolved in H_2_O), 5-BDBD (100 μM, dissolved in DMSO), and AZ 11645373 (10 μM, dissolved in DMSO), as well as antagonist of pannexin channels—CBX (10 μM, dissolved in H_2_O) or thapsigargin (THAPS, 10 nM, dissolved in DMSO)—inhibitor of the sarco/endoplasmic reticulum Ca^2+^ ATPase (SERCA) for appropriate time points. Appropriate solvent was added to respective controls.

### Animals

All the experiments were carried out on male Wistar rats, 4 months old, supplied from Animal House of Medical Research Center, Polish Academy of Sciences (Warsaw, Poland), which operates breeding of small rodents in SPF standard. The animals were maintained under controlled conditions of temperature and humidity with 12-h light/dark cycle. All efforts were made to minimize animal suffering and to reduce the number of animals used. All manipulations were performed gently and quickly to avoid stress-induced alterations.

### Preparation of synaptoneurosomes-enriched fraction

**Fig. 1:**
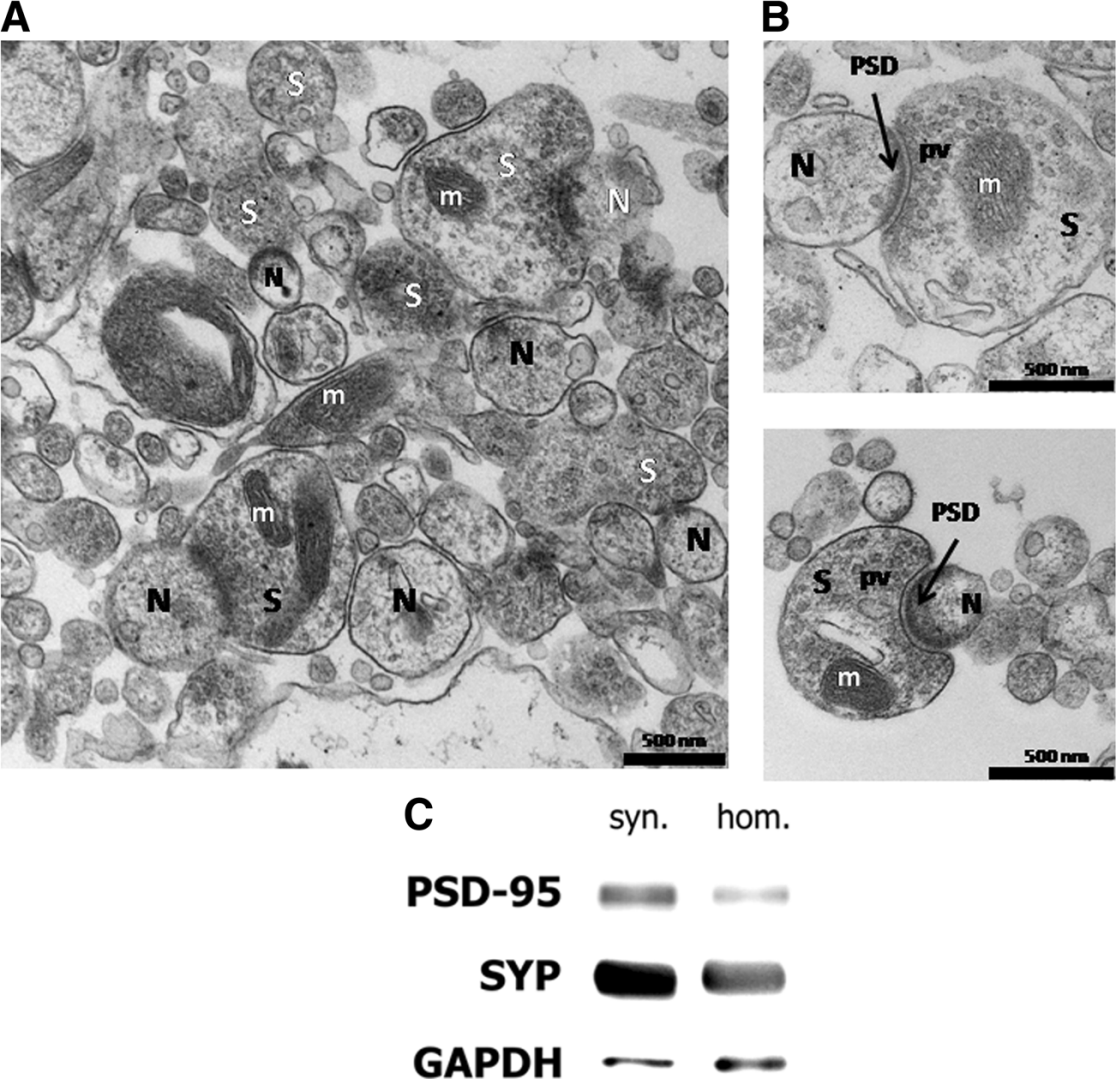
Electron micrographs of representative sample of synaptoneurosomes-enriched fraction isolated from mouse cerebral cortex and hippocampus. **a** A typical large field view of synaptoneurosomes (8.000×). Mitochondria (*m*) are also present. **b** Higher magnification of the complete synaptoneurosome where the presynaptic terminal displays vesicles and a postsynaptic membrane contains PSDs (20.000×). Synaptosome (*S*), presynaptic vesicles (*pv*), neurosome (*N*), and densely stained membranes that characterize PSDs (*PSD*) are shown. **c** Total protein (40 μg) from rat synaptoneurosomes-enriched fraction (*syn.*) or brain tissue homogenates (*hom.*) was analyzed by Western blot. The pre- and postsynaptic protein markers evaluated include synaptophysin and postsynaptic density protein 95 (*PSD95*). *GAPDH* served as a loading control

Male Wistar rats were decapitated, brains were isolated, and the cerebral cortex, including the hippocampus, was manually dissected with a chilled razor blade on an ice-chilled Petri dish. Synaptoneurosomes were prepared according to Adamczyk and Strosznajder [10] and Strosznajder and Samochocki [35]. Briefly, brain slices were homogenized by hand (five strokes) in 7 ml Krebs–Henseleit buffer, pH 7.4 [120 mM NaCl, 5 mM KCl, 1.2 mM MgSO_4_, 1.2 mM KH_2_PO_4_, 25 mM NaHCO_3_, and 10 mM glucose equilibrated with O_2_/CO_2_ (95/5)], using a Dounce-type glass homogenizer. The homogenate was diluted with 28 ml Krebs–Henseleit buffer and centrifuged at 1100×*g* for 15 min. The supernatant was decanted, and the pellet was resuspended in 5 ml of the HBSS solution with 5 mM, *N*-(2-hydroxyethyl)piperazine-*N*′-(2-ethanesulfonic acid) (HEPES) buffer of pH 7.4. The particulate preparations from rat cerebral cortex were examined by electron microscopy as described previously [35]. The preparation contained erythrocytes, axonal fragments, dendritic processes, cell nuclei, myelin fragments, and free mitochondria, but the majority of the content were presynaptic sacs (synaptosomes) attached to membrane-bound postsynaptic sacs (neurosomes) (Fig. 1a, b). Further, Western blot analysis of the obtained fraction showed that this preparation is more characterized with the enrichment of pre- and postsynaptic proteins: synaptophysin and postsynaptic density protein 95 (PSD95), compared to homogenate from brain cortex (Fig. 1c). For further experiments, the obtained preparation rich with synaptoneurosomes was preincubated at 37 °C for 30 min.

### Fluorometric measurements of changes in [Ca^2+^]_*i*_

Changes in intracellular Ca^2+^ ([Ca^2+^]_*i*_) concentration in SH-SY5Y cells were monitored using the fluorescent calcium-sensitive probe Fluo-4. Its acetoxymethyl ester derivative, Fluo-4 AM, easily penetrates plasma membranes, and inside the cells, it is cleaved by esterases to Fluo-4, which becomes highly fluorescent after binding with Ca^2+^. The procedure is essential, as has been described previously by Wilkaniec et al. [36]. SH-SY5Y cells were seeded onto collagen-coated 96-well dark plates at the density of 1.4 × 10^5^ cells/ml. After 24 h, the cells were loaded with 10 μM Fluo-4 AM supplemented with 0.02% Pluronic® F-68 for 60 min at 37 °C in a standard HBSS. The cells were washed three times with HBSS and, to ensure complete AM ester hydrolysis, kept for 30 min at 37 °C in the dark. After a second washing, the fluorescence was measured using a microplate reader FLUOstar Omega (Ortenberg, Germany) set at 485 nm excitation and 538 nm emission wavelengths. After determining the baseline fluorescence of the cells incubated in HBSS, the changes in fluorescence after the addition of the test compounds were recorded every 15 s for 5 min. This 5-min treatment did not have any significant impact on cell viability. The results of fluorescence measurements are presented as percent changes in fluorescence intensity relative to the basal level versus duration of measurement (%F/F0). To quantify the change in the dynamics of the Ca^2+^ responses, the area under the curve (AUC) was calculated as a measure for the increase in intracellular Ca^2+^ [37].

### Assay of radioactive ^[45]^Ca^2+^ influx in neuroblastoma cell line

The procedure was performed as described previously [38]. The SH-SY5Y cells (4 × 10^6^ cells/well) were preincubated for 5 min in the Locke 5 medium containing 5 mM HEPES (pH 7.4), followed by a 5-min incubation with appropriate agents. Then, ^[45]^CaCl_2_ (1 μCi/well) was added for 10 min. The incubation was terminated by washing with ice-cold calcium-free medium containing 2 mM ethylene glycol-bis(β-aminoethyl ether)-*N*,*N*,*N*′,*N*′-tetra acetic acid (EGTA), and the cells were dissolved in 0.5 M NaOH for 30 min at 4 °C. The content of radioactive ^[45]^ Ca^2+^ in the cultures was measured by liquid scintillation spectroscopy using a Wallac 1409 counter (Wallac, Turku, Finland).

### Assay of radioactive ^[45]^Ca^2+^ influx in synaptoneurosome-enriched fraction

The radiochemical measurement of ^[45]^Ca^2+^ influx was performed according to Adamczyk, Strosznajder [10] with slight modifications. Briefly, 0.7 mg of the synaptoneurosomes preparation in 0.1 ml HBSS containing 1.2 mM CaCl_2_ was preincubated at 37 °C for 30 min either alone or with ASN (10 μM) or ATP (5 mM). In selected experiments, synaptoneurosomes were first preincubated with PPADS (100 μM) or AZ 11645373 (10 μM) for 5 min before the addition of ASN or ATP. The reaction was started by adding 0.1 μCi ^[45]^CaCl_2_. After 30 s of incubation at 37 °C, the samples were diluted with 5 ml ice-cold assay buffer with 5 mM EGTA, immediately filtered through Whatman GF/C filters under vacuum pressure, and washed three times in 5 ml assay buffer. Filters were placed in 8 ml Bray’s scintillation fluid, and intrasynaptoneurosomal ^[45]^Ca^2+^ was measured in a liquid scintillation counter LKB Wallac 1409 (Finland).

### RT-PCR

The total RNA isolation was performed according to the procedure developed by Chomczyński, using TRI Reagent® (cat. T9424) from Sigma-Aldrich, following the manufacturer’s protocol. Digestion of DNA contamination was performed using DNase I according to the manufacturer’s protocol (Sigma-Aldrich, St. Louis, MO, USA). RNA quantity and quality were controlled by spectrophotometric analysis and gel electrophoresis. A reverse transcription was performed by using the high capacity cDNA reverse transcription kit according to the manufacturer’s protocol (Applied Biosystems, Foster City, CA, USA). Quantitative real-time PCR was performed with TaqMan Universal PCR Master Mix (Applied Biosystems, Foster City, CA, USA) and detected by a Real-Time PCR System on an ABI PRISM 7500 apparatus (Thermo Fisher Scientific, Waltham, MA, USA) using the commercially available TaqMan® Gene Expression Assays: Hs00602442_m1 (*P2rx4*, amplicon length 91), Hs00175721_m1 (*P2rx7*, amplicon length 89), Hs00704965_s1 (*P2ry1,* amplicon length 73), Hs04176264_s1 (*P2ry2*, amplicon length 82), and Hs01060665_g1 (*Actb*, amplicon length 63). After 2-min incubation at 50 °C, required for optimal AmpErase UNG activity followed by activation of AmpliTaq Gold Enzyme (10 min incubation at 95 °C), a standard two-step PCR amplification was performed, with a melting step at 95 °C for 15 s and annealing and elongation at 60 °C for 1 min, for 40 cycles. *Actb* was used in the analysis as a reference gene. The relative levels of target messenger RNA (mRNA), normalized to an endogenous reference and relative to a calibrator, were calculated by 2^−ΔΔCT^ formula.

## Western blot analysis

The cells were washed three times with ice-cold PBS and lyzed in cell lysis buffer (1×). Protein levels were determined using the Lowry method, and then the samples were mixed with Laemmli buffer and denatured at 95 °C for 5 min. After standard 10% SDS-PAGE separation, proteins were transferred onto PVDF membranes at 100 V. Next, the membranes were washed for 5 min in 100 mM Tris-buffered saline with 0.1% Tween 20 (TBST) and 140 mM NaCl at pH 7.6, and the non-specific bindings were blocked for 60 min at room temperature (RT) with 5% BSA solution in TBST or with 5% non-fat milk solution in TBST. Further, membranes were washed three times for 5 min in TBST and incubated with the following primary antibody: rabbit monoclonal anti-P2X7R (Sigma-Aldrich, cat. P8232; 1∶200) [39] in a 5% BSA solution in TBST, overnight at 4 °C and rabbit monoclonal anti-P2Y1 (Sigma-Aldrich, cat. P6487; 1∶200) [40] in TBST overnight at 4 °C. Then, the membranes were washed three times (5 min) in TBST and incubated for 60 min at RT with secondary antibody (anti-rabbit or anti-mouse IgG) (1∶4000) in a 5% non-fat milk/TBST. Antibodies were detected using chemiluminescent Clarity Western ECL Substrate (Bio-Rad Laboratories, Hercules, CA, USA) under standard conditions. After stripping, the immunolabeling of GAPDH was performed as a loading control.

### Measurement of extracellular pools of ATP

Measurement of ATP level was performed according to the method previously described by Karczewska et al. [41]. For the determination of extracellular ATP concentration, SH-SY5Y cells were seeded onto collagen-coated 24-well plates at the density of 1.5 × 10^5^ cells/ml. After 24 h, the culture medium was carefully changed into 300 μl of HBSS with 5 mM HEPES (pH 7.4) and cells were preincubated for 60 min at 37 °C. Then, the selected agents were carefully added to the cells for 1 min. Later, 100 μl of incubation media was collected into Eppendorf tubes placed at 99 °C for 2 min and centrifuged (4 °C, 800×*g*, 10 min), in order to inactivate the enzymatic degradation processes (quenching) that occurs during sample preparation and to ensure the stability of ATP [42]. Immediately, prior to measurement, the supernatants were brought to ambient temperature (∼23 °C) and the ATP concentration from each sample aliquot was measured by luminometry using Molecular Probes® ATP Determination Kit. This assay based on luciferase’s absolute requirement for ATP in producing light is extremely sensitive: it enables the detection of as little as 0.1 picomole of preexisting ATP. Luminescence was measured in 100 μl reaction mixture containing 500 μM luciferin and 1.25 μg/ml luciferase at room temperature with microplate reader FLUOstar Omega (Ortenberg, Germany).

### Determination of ecto-ATPase activity

For determination of ecto-ATPase activity, hydrolysis of exogenously added ATP was determined. SH-SY5Y cells were seeded onto collagen-coated 24-well plates at the density of 1.5 × 10^5^ cells/ml. After 24 h, the culture medium was carefully changed into 300 μl of HBSS with 5 mM HEPES (pH 7.4) and cells were preincubated for 60 min at 37 °C. Then, ASN (10 μM) was carefully added to the cells. Incubation with ASN was performed in the presence and absence of 50 μM ARL 67156, a selective ecto-ATPase inhibitor, in order to check the specific ecto-ATPase-dependent ATP degradation. The assay was initiated by addition of ATP (100 μM) to SH-SY5Y cells. After 15 min of incubation at 37 °C, 100 μl of incubation media was collected into Eppendorf tubes, placed at 99 °C for 2 min, and centrifuged (4 °C, 800×*g*, 10 min). Immediately prior to measurement, the supernatants were brought to ambient temperature (∼23 °C) and the ATP concentration from each sample aliquot was measured by luminometry using Molecular Probes® ATP Determination Kit. Specific ATP degradation by ecto-ATPase was calculated by subtracting the results obtained without ARL 67156 from the ones determined in the presence of ARL 67156. The data were presented as micromole ATP per minute.

### Statistical analysis

The results that were expressed as mean values ± Standard Error of Mean (SEM); differences between the means were analyzed using a Student’s *t* test between two groups or one-way analysis of variance (ANOVA) with Bonferroni multiple comparison post-hoc test among multiple groups. Statistical significance was accepted at *p* < 0.05. The statistical analyzes were performed using GraphPad Prism version 4.0 (GraphPad Software, San Diego, CA).

## Results

Since extracellular ASN was previously shown to induce changes in purinergic receptors expression and activity in microglial cells [12], we first verified whether this protein affects mRNA and protein level, as well as the activity of selected P2 receptors subtypes in neuronal cells. We used human neuroblastoma SH-SY5Y cell line, and because these cells are able to express a number of features characteristic for catecholaminergic neurons, including tyrosine hydroxylase and dopamine-β-hydroxylase activities [43], they express various P2 receptors belonging to both P2X and P2Y families as well [44]. From the whole range of purinergic P2X ionotropic and P2Y metabotropic receptors, we have chosen to study few that were previously shown to be connected with neurodegeneration [13].

**Fig. 2:**
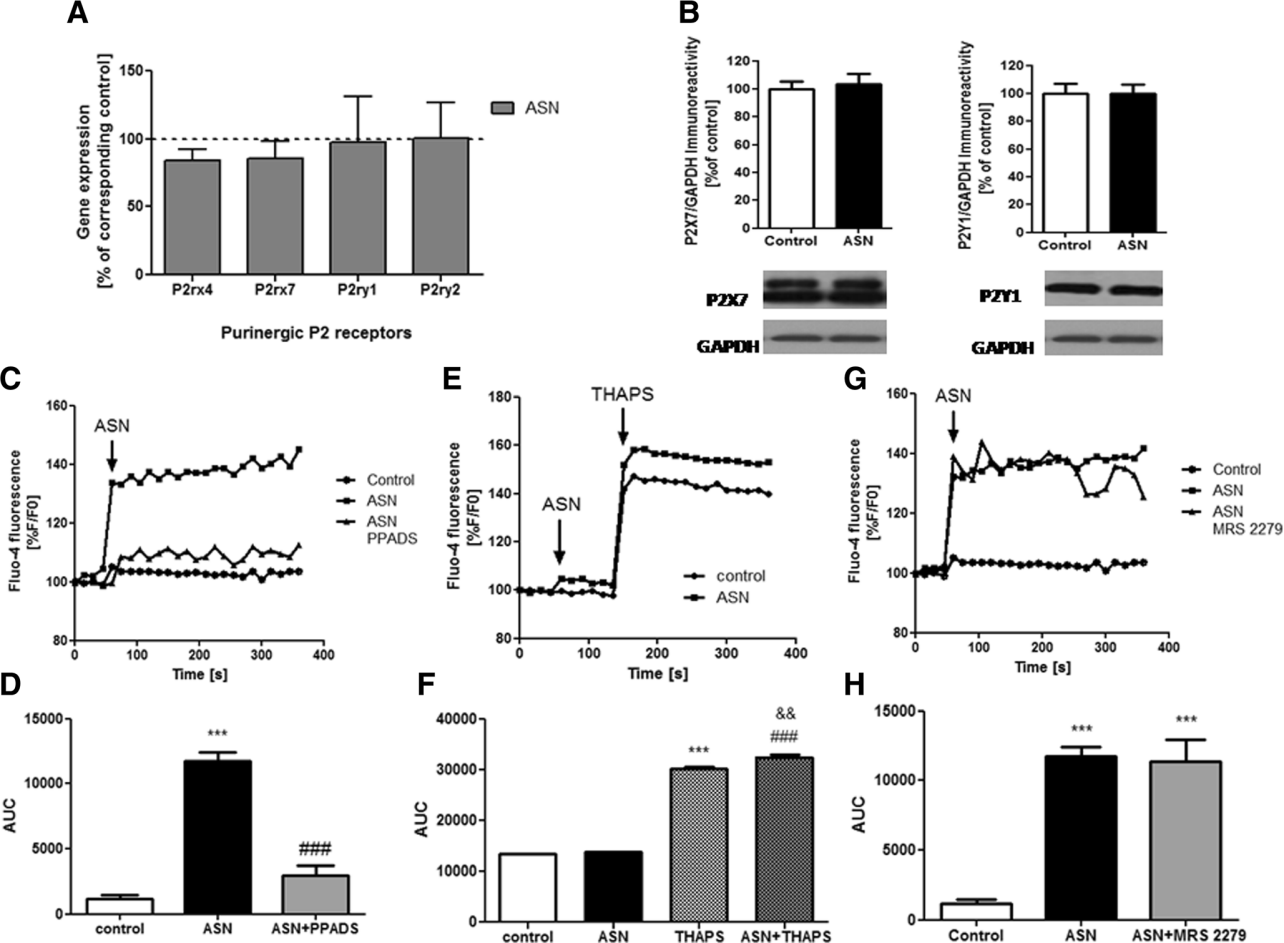
The effect of extracellular ASN treatment on the expression and activity of purinergic P2 receptors. **a** Gene expression for P2 receptors was analyzed by quantitative RT-PCR after 24 h treatment with ASN (10 μM) in culture medium containing 2% FBS. Data are expressed as a percent of corresponding control and represent the mean value ± SEM for three separate experiments (*n* = 3). **b** Immunoreactivity of P2X7 and P2Y1 receptors was analyzed by SDS-PAGE and Western blotting after 24 h treatment with ASN (10 μM) in culture medium containing 2% FBS. Representative pictures were shown. Results of densitometric analysis were normalized to immunoreactivity of GAPDH, as a loading control. The results are presented as the mean ± SEM from seven independent experiments (*n* = 7). **c** SH-SY5Y cells were preincubated for 60 s with 100 μM PPADS (P2 receptors antagonist) in HBSS. Baseline Fluo-4 fluorescence was monitored for 60 s prior to the addition of ASN (10 μM). Fluorescence was monitored for a further 300 s and values were converted to %F/F0, where F0 is the fluorescence value of first record (0 s). **d** Responses were quantitated by measuring the AUC value. The results are presented as the mean ± SEM from six independent experiments (*n* = 6). ***p < 0.001 versus control, ^###^ *p* < 0.001 versus ASN-treated cells using a one-way ANOVA followed by the Bonferroni test. **e** SH-SY5Y cells were preincubated for 60 s in calcium-free HBSS buffer containing 10 mM Tris and 1 mM EGTA. Baseline Fluo-4 fluorescence was monitored for 60 s prior to the addition of ASN (10 μM). Fluorescence was monitored for a further 90 s (first peak). Then, the calcium influx (second peak) was initiated by the addition of THAPS (10 nM). Values were converted to %F/F0, where F0 is the fluorescence value of first record (0 s). **f** Responses were quantitated by measuring the AUC value of first and second peak. The results are presented as the mean ± SEM from three independent experiments (*n* = 3). ***p < 0.001 versus control, ^###^ *p* < 0.001 versus ASN-treated cells, ^&&^ *p* < 0.01 versus THAPS-treated cells using a one-way ANOVA followed by the Bonferroni test. **g** SH-SY5Y cells were preincubated for 60 s with 10 μM MRS 2279 (P2Y1 receptors antagonist) in HBSS. Baseline Fluo-4 fluorescence was monitored for 60 s prior to the addition of ASN (10 μM). Fluorescence was monitored for a further 300 s and values were converted to %F/F0, where F0 is the fluorescence value of first record (0 s). **h** Responses were quantitated by measuring the AUC value. The results are presented as the mean ± SEM from three to six independent experiments (*n* = 3–6). ***p < 0.001 versus control using a one-way ANOVA followed by the Bonferroni test

In the current study, we observed that a 24-h treatment with exogenous ASN (10 μM) did not change the mRNA level of the P2X4, P2X7, P2Y1, and P2Y2 receptors (Fig. 2a), as well as had no influence on the immunoreactivity of selected P2X7 and P2Y1 receptors (Fig. 2b). In order to investigate the functional impact of ASN treatment on P2X receptors coupled to activation of Ca^2+^ channels, we used PPADS, a widely used non-selective P2X receptor antagonist. Treatment of SH-SY5Y cells with 10 μM ASN resulted in significant [Ca^2+^]_*i*_ mobilization and pretreatment with 100 μM PPADS for 2 min, followed by exposure to ASN, almost completely abolished the effect of this protein on [Ca^2+^]_*i*_ (Fig. 2c, d). In order to determine whether the increase of [Ca^2 +^]_*i*_ is related to metabotropic P2Y receptor-mediated Ca^2+^ release from endoplasmic reticulum (ER), SH-SY5Y cells were treated with ASN in a calcium-free medium containing additional Ca^2+^-chelator, EGTA. In those experimental conditions, we observed that exogenous ASN had no impact on [Ca^2+^]_*i*_ mobilization. Moreover, depletion of ER Ca^2+^ stores with 10 nM THAPS, a sarco/endoplasmic reticulum Ca^2+^ ATPase (SERCA) inhibitor, led to a significant enhancement of [Ca^2+^]_*i*_ level in SH-SY5Y cells treated with ASN, compared to untreated cells (Fig. 2e, f). Also, SH-SY5Y cells pretreated with selective P2Y1 receptor antagonist, MRS 2279 (10 μM) did not reverse ASN-evoked changes in [Ca^2+^]_*i*_ mobilization and were comparable to the effects of ASN treatment alone (Fig. 2g, h). These data suggest that P2X-mediated Ca^2+^ influx, but not P2Y-related ER stores mobilization, is involved in [Ca^2+^]_*i*_ increase after ASN treatment.

**Fig. 3:**
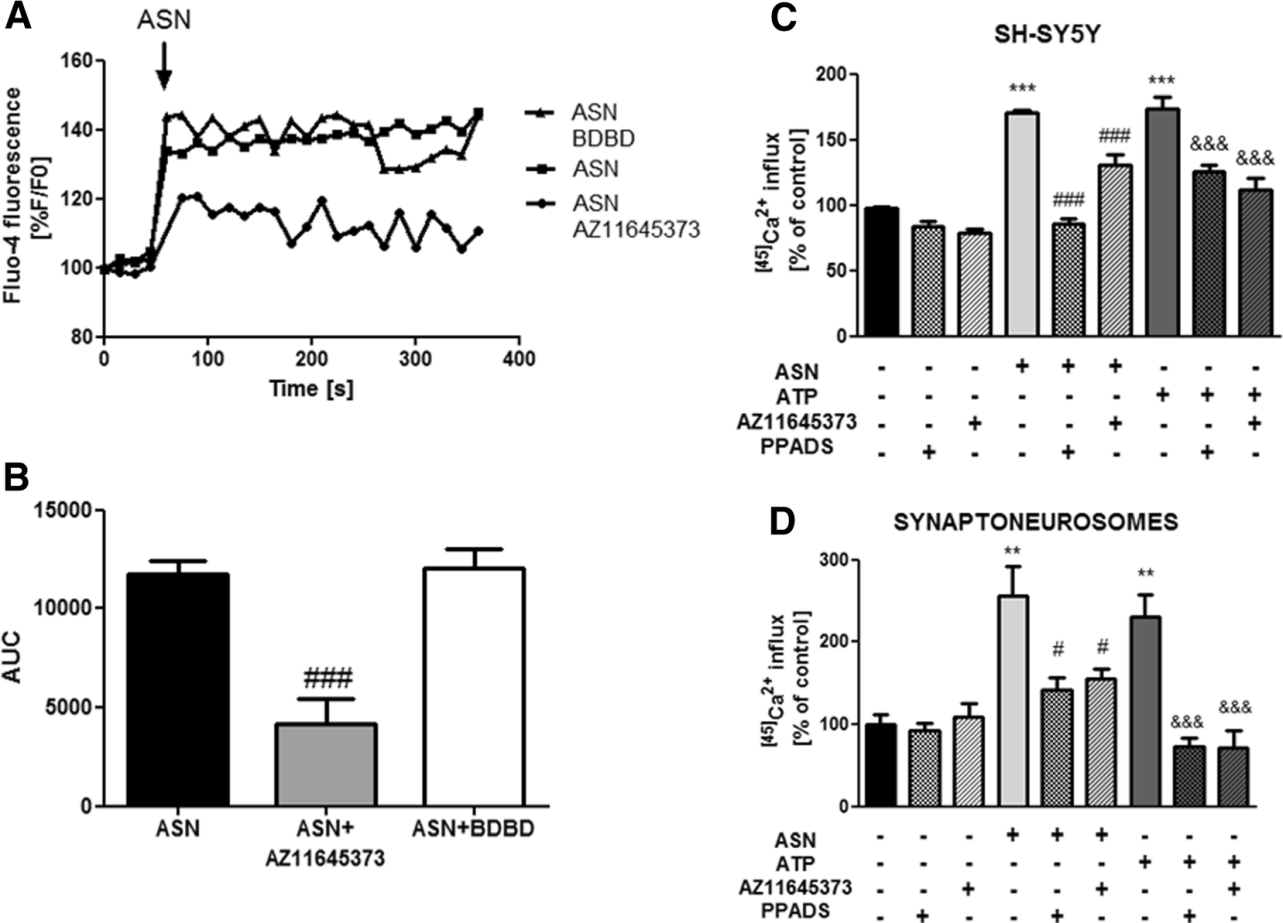
P2X7, but not P2X4 receptor, mediates ASN-induced calcium influx. **a** SH-SY5Y cells were preincubated for 60 s with 10 μM AZ 11645373 (P2X7 receptors antagonist) or 100 μM 5-BDBD (P2X4 receptor antagonist) in HBSS. Baseline Fluo-4 fluorescence was monitored for 60 s prior to the addition of ASN (10 μM). Fluorescence was monitored for a further 300 s, and values were converted to %F/F0, where F0 is the fluorescence value of first record (0 s). **b** Responses were quantitated by measuring the AUC value. The results are presented as the mean ± SEM from three to six independent experiments (*n* = 3–6). ^###^ *p* < 0.001 versus ASN-treated cells using a one-way ANOVA followed by the Bonferroni test. **c** The SH-SY5Y cells were preincubated with 10 μM AZ 11645373 or 100 μM PPADS for 5 min in the Locke 5 medium containing 5 mM HEPES (pH 7.4), followed by a 5 min incubation with 10 μM ASN or 5 mM ATP. Then, ^[45]^CaCl_2_ (1 μCi/well) was added for 10 min. The results are presented as the mean ± SEM from three to six independent experiments (*n* = 3–6). ***p < 0.001 versus control, ^###^ *p* < 0.001 versus ASN-treated cells, ^&&&^ *p* < 0.001 versus ATP-treated cells using a one-way ANOVA followed by the Bonferroni test. **d** Rat brain synaptoneurosomes were preincubated with PPADS (100 μM) or AZ 11645373 (10 μM) for 5 min, followed by a 30-min incubation with 10 μM ASN or 5 mM ATP at 37 °C in HBSS medium (pH 7.4). The reaction was started by adding 0.1 μCi ^[45]^CaCl_2_. The results are presented as the mean ± SEM from three to six independent experiments (*n* = 3–6). **p < 0.01 versus control, ^#^ *p* < 0.05 versus ASN-treated cells, ^&&&^ *p* < 0.001 versus ATP-treated cells using a one-way ANOVA followed by the Bonferroni test

Among other P2X receptors expressed by SH-SY5Y cells, we focused on P2X4 and P2X7, because they share some functional features and were previously shown to be involved in apoptosis of neuronal cells [33, 45–47]. We analyzed the influence of selective antagonists of P2X7 (AZ 11645373) and P2X4 (5-BDBD) receptors on ASN-induced [Ca^2+^]_*i*_ mobilization. Pretreatment with 10 μM AZ 11645373 almost completely abolished the effect of ASN on [Ca^2+^]_*i*_ (Fig. 3a), whereas the effect of 5-BDBD was negligible (Fig. 3b), suggesting that the observed [Ca^2 +^]_*i*_ changes were mediated by activation of P2X7 receptor. For further evidence of the involvement of P2X7 receptor in the fast [Ca^2+^]_*i*_ mobilization, induced by ASN, we performed the measurements of ^[45]^Ca^2+^ uptake, which reflects the influx of extracellular Ca^2+^ into the cells. A P2X7 receptor stimulation, evoked by the addition of 5 mM ATP, produced a significant increase in ^[45]^Ca^2+^ influx. Application of 10 μM ASN potentiated the ^[45]^Ca^2+^ uptake to 170% of the control, and this effect was comparable with the same observed for ATP. Moreover, both non-selective (PPADS) and selective (AZ 11645373) antagonists of P2X7 receptor significantly reduced the effect of ASN and ATP treatment on ^[45]^Ca^2+^ influx (Fig. 3c). To confirm that the observed phenomena are not only characteristic for cultured cells of tumor origin, we conducted the ex vivo experiments in synaptic nerve ending-enriched fraction isolated from rat brain. The results were similar to those observed in SH-SY5Y cells and showed that pretreatment with AZ 11645373 or PPADS prevented ^[45]^Ca^2+^ influx induced by either ASN or ATP (Fig. 3d), thus conclusively evidenced the participation of P2X7 in the Ca^2+^ imbalance caused by exogenous ASN.

**Fig. 4:**
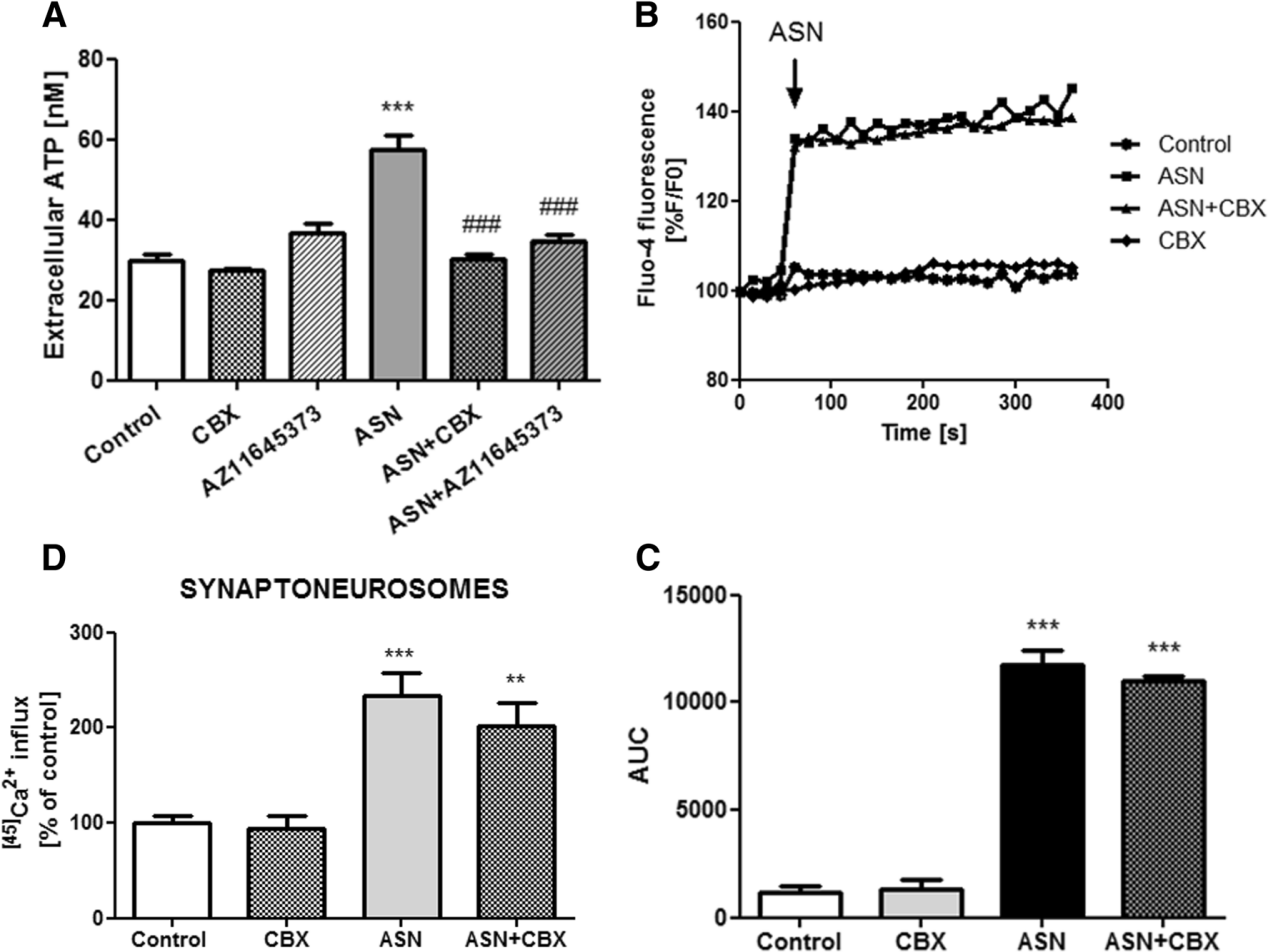
Activation of P2X7 receptors leads to pannexin 1-dependent ATP release. **a** Extracellular ATP level was analyzed after 1-min treatment with 10 μM ASN together or without 10 μM CBX (antagonist of pannexin channels) or 10 μM AZ 11645373 (P2X7 receptors antagonist) at 37 °C in HBSS with 5 mM HEPES (pH 7.4) using the luciferase-based protocol described in the “Materials and methods” section. Data, calculated from respective calibration curves, are expressed as nM ATP and the data represent the mean value ± SEM for six to eight separate experiments (*n* = 6–8). ***p < 0.001 versus control, ^###^ *p* < 0.001 versus ASN-treated cells using a one-way ANOVA followed by the Bonferroni test. **b** SH-SY5Y cells were preincubated (60 s) with 10 μM CBX or appropriate solvent in HBSS. Baseline Fluo-4 fluorescence was monitored for 60 s prior to the addition of ASN (10 μM) or appropriate solvent. Fluorescence was monitored for a further 300 s, and values were converted to %F/F0, where F0 is the fluorescence value of first record (0 s). **c** Responses were quantitated by measuring the AUC value. The results are presented as the mean ± SEM from four to six independent experiments (*n* = 4–6). ***p < 0.001 versus control using a one-way ANOVA followed by the Bonferroni test. **d** Rat brain synaptoneurosomes were preincubated with CBX (10 μM) for 5 min, followed by a 30-min incubation with 10 μM ASN at 37 °C in HBSS medium (pH 7.4). The reaction was started by adding 0.1 μCi ^[45]^CaCl_2_. The results are presented as the mean ± SEM from three to six independent experiments (*n* = 3–6). **p < 0.01, ***p < 0.001 versus control using a one-way ANOVA followed by the Bonferroni test

**Fig. 5:**
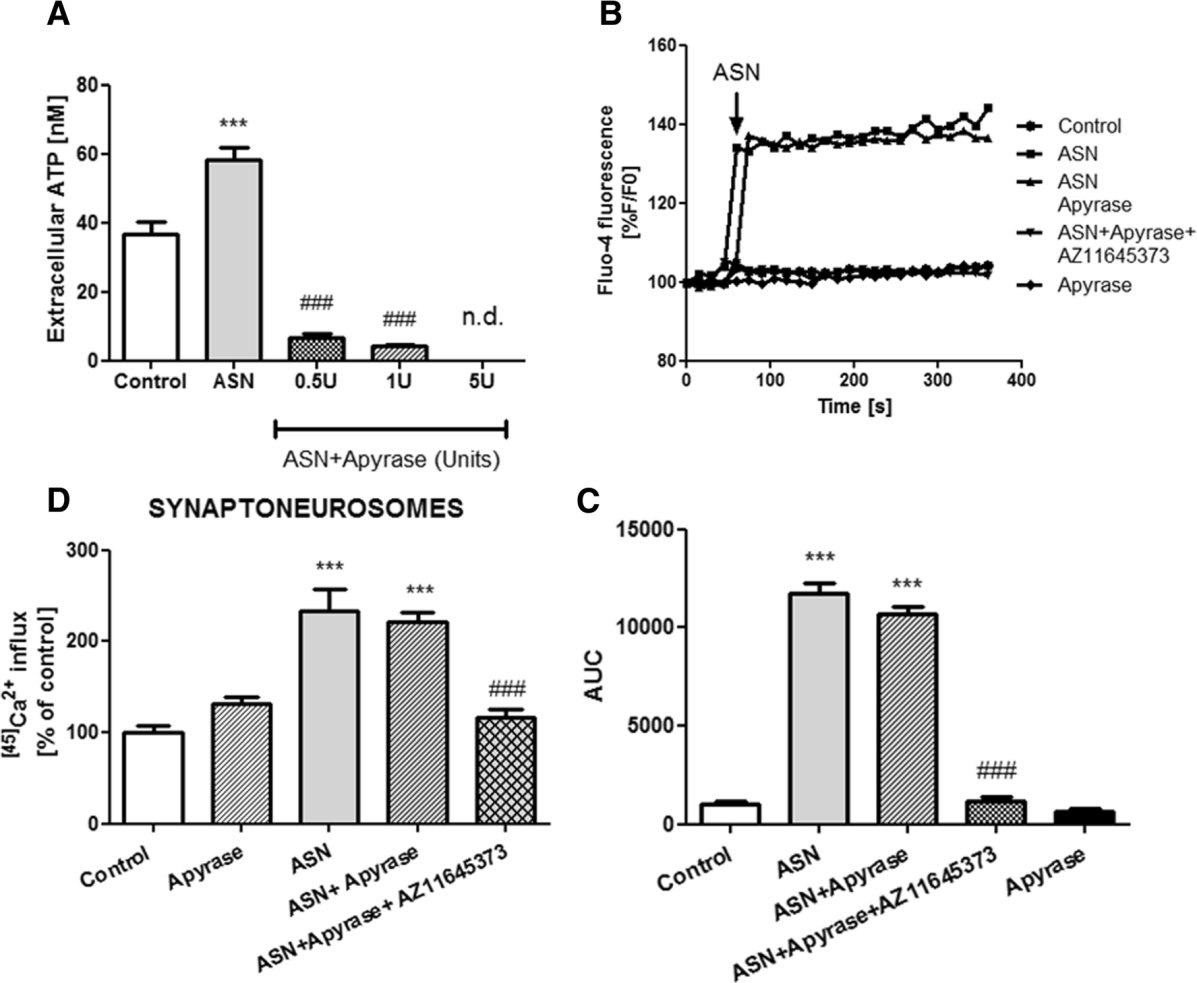
The increase in extracellular ATP by ASN is not responsible for P2X7 receptor activation. **a** Extracellular ATP level was analyzed after 1-min treatment with 10 μM ASN together or without 0.5, 1, or 5 U apyrase at 37 °C in HBSS with 5 mM HEPES (pH 7.4) using the luciferase-based protocol described in the “Materials and methods” section. Data, calculated from respective calibration curves, are expressed as nM ATP and the data represent the mean value ± SEM for three separate experiments (*n* = 3). ***p < 0.001 versus control, ^###^ *p* < 0.001 versus ASN-treated cells using a one-way ANOVA followed by the Bonferroni test. **b** SH-SY5Y cells were preincubated (60 s) with 1 U apyrase with or without 10 μM AZ 11645373 (P2X7 receptors antagonist) or appropriate solvent in HBSS. Baseline Fluo-4 fluorescence was monitored for 60 s prior to the addition of ASN (10 μM) or appropriate solvent. Fluorescence was monitored for a further 300 s, and values were converted to %F/F0, where F0 is the fluorescence value of first record (0 s). **c** Responses were quantitated by measuring the AUC value. The results are presented as the mean ± SEM from four independent experiments (*n* = 4). ***p < 0.001 versus control, ^###^ *p* < 0.001 versus ASN-treated cells using a one-way ANOVA followed by the Bonferroni test. **d** Rat brain synaptoneurosomes were preincubated with 1 U apyrase with or without 10 μM AZ 11645373 for 5 min, followed by a 30-min incubation with 10 μM ASN at 37 °C in HBSS medium (pH 7.4). The reaction was started by adding 0.1 μCi ^[45]^CaCl_2_. The results are presented as the mean ± SEM from three independent experiments (*n* = 3). ***p < 0.001 versus control, ^###^ *p* < 0.001 versus ASN-treated cells using a one-way ANOVA followed by the Bonferroni test

Considering the controlling mechanisms of P2X7 receptor activation, ASN-mediated release of ATP from the cells is possibly involved in the P2X7-mediated deregulation of [Ca^2+^]_*i*_. Our biochemical experiments showed that 1-min treatment with exogenous ASN increased almost twofold the amount of extracellular ATP (Fig. 4a). Upon a variety of stimulations, ATP can be released by membrane transporters, particularly, pannexin channels (Panx) [48]. To block ATP release from cells, we used carbenoxolone (CBX; 10 μM), a selective inhibitor of Panx1, and observed that this compound normalized extracellular ATP levels in SH-SY5Y cells treated with ASN. P2X7 receptor blockade with AZ 11645373 also resulted in significant reduction of the extracellular ATP release after ASN treatment (Fig. 4a). Moreover, pretreatment with CBX had no effect on either elevation of [Ca^2+^]_*i*_ in SH-SY5Y cells (Fig. 4b, c) or ^[45]^Ca^2+^ influx in rat brain synaptoneurosomes (Fig.4d) induced by ASN. This indicated that ATP release by Panx is not responsible for P2X7 receptor activation induced by ASN treatment. To further confirm that ASN could directly affect the P2X7 receptor function, we treated the cells with apyrase (EC 3.6.1.5), a calcium-activated enzyme that catalyzes the hydrolysis of ATP to AMP and inorganic phosphate. We observed that in the presence of apyrase, the extracellular ATP level was almost completely reduced in SH-SY5Y cells treated with ASN, and this decrease was proportional to the applied dose of apyrase (Fig. 5a). In the conditions of 90% extracellular ATP withdrawal (pretreatment with apyrase at the dose of 1 U), the effect of exogenous ASN on [Ca^2+^]_*i*_ mobilization in SH-SY5Y cells was similar to this observed in the absence of apyrase (Fig. 5b, c). Moreover, in the presence of apyrase, AZ 11645373 was still able to reduce the effect of ASN treatment on [Ca^2+^]_*i*_ (Fig. 5b, c). Similar results were observed in ex vivo experiments in synaptic nerve endings and showed that the significant reduction of extracellular ATP did not change the effect of ASN on ^[45]^Ca^2+^ influx in a manner prevented by AZ 11645373 (Fig. 5d), thus conclusively evidenced the direct interaction of exogenous ASN with P2X7 receptor.

**Fig. 6:**
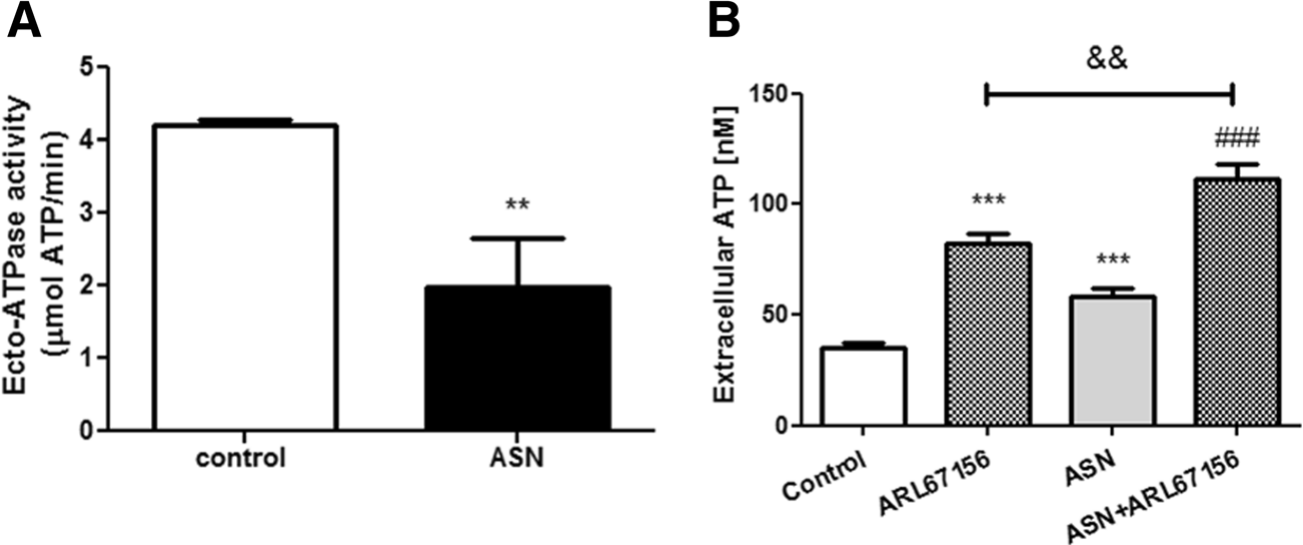
Extracellular ASN decreases the activity of ecto-ATPase in SH-SY5Y cells. **a** Hydrolysis of exogenously added ATP (100 μM) by the SH-SY5Y cells treated for 10 min with 10 μM ASN in the presence/absence of 50 μM ARL 67156 (ecto-ATPase inhibitor) was determined by luminometry analysis described in the “Materials and methods” section. Data, calculated from respective calibration curve, are expressed as μM of hydrolyzed ATP per min and they represent the mean value ± SEM for three separate experiments (*n* = 3). **p < 0.01 versus control by Student’s *t* test. **b** Extracellular ATP level was analyzed after 1-min treatment with 10 μM ASN together or without 50 μM ARL 67156 at 37 °C in HBSS with 5 mM HEPES (pH 7.4) using the luciferase-based protocol described in the “Materials and methods” section. Data, calculated from respective calibration curves, are expressed as nM ATP and the data represent the mean value ± SEM for three separate experiments (*n* = 3). ***p < 0.001 versus control, ^###^ *p* < 0.001 versus ASN-treated cells, ^&&^ *p <* 0.01 versus ARL67156-treated cells using a one-way ANOVA followed by the Bonferroni test

Finally, we observed that ASN significantly decreased the activity of the ecto-adenosine triphosphatase (ecto-ATPase, EC 3.6.1.3), the major plasma membrane nucleotidase responsible for the hydrolysis of extracellular ATP (Fig. 6a). Moreover, the inhibition of ecto-ATPase with 50 μM ARL 67156 resulted in a significant elevation of extracellular ATP level, and the effect of the ecto-adenosine triphosphatase inhibitor was more pronounced when the cells were treated with ASN (Fig. 6b).

## Discussion

In the present study, we have discussed the pivotal role of extracellular ASN in the deregulation of purinergic signaling. Our data have shown that interaction of ASN with P2X7 receptor is responsible for its activation. These findings are thus consistent with previous studies, which demonstrated that ASN leads to activation of microglial P2X7 receptor [12], and those findings are extended by showing that this important interaction occurs also in neuronal cells.

Since neurodegenerative diseases share common features and mechanisms responsible for inducing the death of selective neuronal populations [49–51], the extracellular release of ASN as well as activation of P2 receptor may likely contribute, alone or in combination, to these mechanisms and to a neurodegenerative process. Purinergic signaling was previously shown to be involved in the etiopathology of many neurodegenerative disorders [52]. Moreover, after metabolic stress, brain ischemia, and trauma, the extracellular ATP and adenosine levels were observed to reach values that were considerably exceeding the ones observed in physiological conditions [14, 53, 54]. Consistently, several P2 receptor antagonists prevented cell death of neurons exposed to oxaliplatin [55], excessive glutamate [56], hypoglycemia [57], and chemical hypoxia [58]. Furthermore, previous studies indicated that an altered activity of the purinergic receptors mediates the proinflammatory processes in a transgenic AD model and in brains from AD patients [27–29] and in vivo inhibition of P2X7 receptors significantly reduces the amyloid plaques formation in brain hippocampal structures through activation of α-secretase activity [59]. Moreover, the recent observation that P2X1 receptor activation induces lysosomal dysfunction and intracellular ASN accumulation puts the role of ATP and P2 receptors as a key event related to misfolded protein response [60]. In the light of these data, we tested whether extracellularly liberated ASN can activate different P2 receptor subtypes, but our observations indicated that P2X7 receptor is exclusively affected by this protein.

Deregulation of P2X7 receptor has previously been indicated to be common for diverse situations of brain damage, such as ischemia [61, 62], traumatic brain injury [63], spinal cord injury [64], epilepsy [65], Alzheimer’s disease [59, 66], Parkinson’s disease [67], prion disease [68], or Huntington’s disease [69]. However, in those pathological conditions, the upregulation of this receptor was observed mainly in glial cells, where P2X7 is ubiquitously expressed [29, 70–75]. Since this receptor was shown to be an obligate participant in microglia activation caused by amyloid beta and ASN [12, 76, 77], the P2X7R-mediated neurotoxicity was connected mostly to the control of the noxious impact of glial cells on neuronal viability. However, recent data also suggest that P2X7R is functionally expressed by neuronal cells and its activation has a direct impact on neuronal cell death [33, 67]. Although our data ultimately excluded the involvement of extracellular ASN in changes of P2X7 receptor expression, they showed that it has a powerful impact on this receptor’s activity in neuronal cells.

Among P2 nucleotide receptors, P2X7 exhibits low sensitivity to ATP, and its maximal activity is achieved with as far as millimolar concentrations [78, 79]. It was initially suggested that the high concentrations of extracellular ATP, required to stimulate cytotoxic actions of this receptor, might not be reached in vivo. However, there is evidence that different noxious brain stimuli are able to substantially increase the extracellular levels of ATP [61, 80–83]. Moreover, damaged neurons are also the source of considerable amounts of released ATP that may act as a “find me” signal for microglial cells [32], which then release excess amounts of reactive oxygen species (ROS), proinflammatory cytokines, etc. [84, 85]. Such high concentrations of ATP existing in pathological conditions also exert direct toxic effect on primary neuronal cultures and organotypic CNS cultures [33]. Therefore, pathophysiological role of extracellular ATP in the regulation of ASN-induced P2X7 receptor dysfunction seemed plausible. In this study, we have observed that substantial increase in ATP secretion from SH-SY5Y cells is associated with ASN treatment. Among several membrane proteins that have been proposed to mediate ATP release, we put special emphasis on the Panx1 channel that does not form functional gap junctions, but is responsible for the release of signaling molecules to extracellular space [86, 87]. Many previous data indicated that Panx1 is expressed in neuronal and epithelial tissues where it mediates ATP release [87]. Recently, it has been reported that Panx1-dependent ATP release from apoptotic cells is a mechanism responsible for phagocyte recruitment [16]. Moreover, Panx1 may indirectly contribute to neuronal death by activating a large protein complex called the inflammasome [88]. Previously, the linkage between P2X7 receptor and Panx1 was also considered to be necessary for P2X7-mediated generation of ROS and proinflammatory cytokines in macrophages [89, 90]. On the other hand, neurons also express both P2X7 and Panx1, but their functionality remains controversial [91–93]. Our current studies have shown that inhibition of Panx1 dampens extracellular ATP levels; however, it does not inhibit P2X7 receptor activity. Moreover, we observed that the selective antagonist of P2X7 receptor is also capable of inhibiting ATP release from SH-SY5Y cells. Those results suggested that P2X7 receptor activation by ASN is responsible for Panx1-dependent ATP release. Indeed, previous reports demonstrated that ATP acting through P2X7 receptor can activate Panx1 channels, enabling the phenomenon of ATP-induced ATP release and thereby amplifying the ATP signal [78]. The mechanism of Panx1 activation by P2X7 receptor has been previously described to be independent of calcium influx [94], but strongly dependent on Src tyrosine kinase activation, following P2X7 receptor stimulation [95]. Moreover, prolonged or repeated activation of Panx1 by P2X7 receptor results in cell death [94], but direct stimulation of Panx1 is not sufficient to induce toxicity per se [96]. Thus, Panx1 appears to be the molecular substrate for the permeabilization pore recruited into the P2X7 signaling complex. Therefore, direct interaction of extracellular ASN with P2X7 subunits, leading to conformational change of the receptor, might be the mechanism responsible for its activation. This hypothesis is evidenced by the previous study that show coimmunoprecipitation of ASN with P2X7 receptor [12]. Moreover, we observed that in the conditions of extracellular ATP withdrawal, the exogenous ASN is still able to activate P2X7 receptor and this effect is reversed by selective inhibitor of P2X7. Based on those results, we can speculate that exogenous ASN can directly activate P2X7 receptor leading to Panx1 recruitment to form an active complex responsible for ATP release in cultured neuronal cells. Though, sustained ATP release from neurons may further autostimulate P2X7 receptors located in the same or neighboring cells, yet the abundance of local nucleotidases can greatly limit the available concentration of ATP by rapidly converting the ATP released from adjacent cells into adenosine [14]. Interestingly, the extracellular catabolism of ATP by ecto-nucleotidases, followed by activation of adenosine receptors, may constitute the indirect impact of ATP on brain dysfunction. This seems to be especially important in the pathology of PD, since a specific synergism between stimulation of D2 receptors and inhibition of adenosine A2A receptors (A2AR) was previously indicated [97]. Moreover, the expression of A2AR in the brain of patients with PD and dyskinesia was increased [98], and in a rat model of PD, adenosine A1, dopamine D1, and glutamate mGlu5 receptors have been shown to interact during locomotion [99]. Followed by those observations, recent data have shown that exogenous ASN treatment triggers selective induction of A2AR expression in both the primary neuronal cells [100] and the animal models of synucleinopathy [101]. In those studies, the genetic deletion of A2AR prevented ASN-induced changes, such as astrogliosis, NF-κB activation, apoptotic neuronal cell death, and impairment of LTP, and prevented from the memory deficits. Intriguingly, blockade of A2AR increased oligomerization of ASN [101], but at the same time, it reduced percentage of cells displaying ASN inclusions [100]. Taken together, those observations may suggest that ASN-induced sustained ATP release from neuronal cells, followed by its extracellular catabolism to adenosine, may result in a parallel bolstering of the ATPergic and adenosinergic arms of the purinergic system, leading to stimulation of cytotoxic effects of purinergic signaling on neighboring microglia, astrocytes, and neurons [14]. Interestingly, our recent observations showed that extracellular ASN greatly decreases the activity of ecto-ATPase that, on one hand, may result in the increase of extracellular ATP content reaching to millimolar concentration, but on the other, this may substantially decrease the formation of ATP-derived adenosine. Since abundance of adenosine is critical for A2AR activation, the obtained results seem to be inconsistent with the overall hypothesis of purinergic deregulation evoked by exogenous ASN. The most probable explanation of this discrepancy might be that the ecto-5′-nucleotidase (CD73, EC 3.1.3.5), the major enzyme able to convert extracellular AMP into adenosine, is more important for the adenosine A2A receptor activation than ecto-ATPase-dependent extracellular ATP catabolism. Indeed, it was demonstrated that CD73 colocalizes with A2AR in the postsynaptic sites of basal ganglia and the CD73 activation is necessary for A2AR-dependent cAMP formation in synaptic terminals [102]. It is also probable that exogenous ASN might inhibit ecto-ATPase while activating CD73; however, this relevant interaction remains to be studied.

In summary, our data is the first to show that interaction between ASN and purinergic P2X7 receptor occurs in neuronal cells. Moreover, our results reveal that P2X7/Panx1-dependent dynamic change of extracellular ATP and inhibition of ATP degradation are key molecular processes involved in extracellular ASN-mediated deleterious signaling. Those new observations, supplemented with the latest studies from other groups, provide the evidence for anti-purinergic therapy that can be an effective treatment of neurodegenerative disorders.
